# Origins of Polynesian Pigs Revealed by Mitochondrial Whole Genome Ancient DNA

**DOI:** 10.3390/ani12182469

**Published:** 2022-09-18

**Authors:** K. Ann Horsburgh, Anna L. Gosling, Ethan E. Cochrane, Patrick V. Kirch, Jillian A. Swift, Mark D. McCoy

**Affiliations:** 1 Department of Anthropology, Southern Methodist University, Dallas, TX 75205, USA; 2 School of Geography, Archaeology and Environmental Studies, University of the Witwatersrand, Johannesburg 2000, South Africa; 3 Department of Anatomy, University of Otago, Dunedin 9016, New Zealand; 4 Anthropology, School of Social Sciences, University of Auckland, Auckland 1010, New Zealand; 5 Department of Anthropology, University of Hawaii at Mānoa, Honolulu, HI 96822, USA; 6 Pacific Legacy, Inc., Kailua, HI 96734, USA

**Keywords:** pig domestication, archaeology, ancient DNA, Polynesia, long-distance voyaging

## Abstract

**Simple Summary:**

Retracing the ancient human migration routes in the remote islands of the Pacific relies on robust models of the origins and spread of animals that were commensal to long-distance ocean voyages. Domestic pigs (*Sus scrofa*) in Polynesia belong to a rare mitochondrial DNA group whose geographic origins are disputed. We report new complete genome ancient DNA that suggests all founding populations of pigs in Polynesia, first settled by people about 2800–700 years ago, can be traced back to northern peninsular Southeast Asia.

**Abstract:**

Domestic pigs (*Sus scrofa*) were first transported to Polynesia through a series of long-distance voyages ultimately linked to the Neolithic expansion of Austronesian-speaking people out of Asia. The descendants of the founding pigs belong to a rare mtDNA group referred to as the “Pacific Clade” that may have originated in peninsular or island Southeast Asia. We report the first whole genome mtDNA from domestic pigs from any of the remote islands of the Pacific. In this brief report, we describe the close link we discovered between ancient mtDNA from archaeological specimens from across Polynesia and from that of modern pigs in northern peninsular Southeast Asia, specifically southern China’s Yunnan Province. More complete mtDNA coverage in commensal animals is necessary to improve our picture of the settlement of Polynesia (ca. 2800–700 years before the present) and specify the route, or routes, that pigs took from northern peninsular Southeast Asia.

## 1. Introduction

The remote islands of the Pacific were the last habitable major region of our planet to be discovered and settled by humans. The settlement of Polynesia—the region’s largest culture area, spread over more than 43 million km^2^—has been reconstructed by triangulating from historical linguistics, oral traditions and ethnography, and archaeology (see [1] for overview). Over the past decades, the study of commensal animals has grown to become foundational to how we test existing models and generate new hypotheses (e.g., [2]).

**Figure 1:**
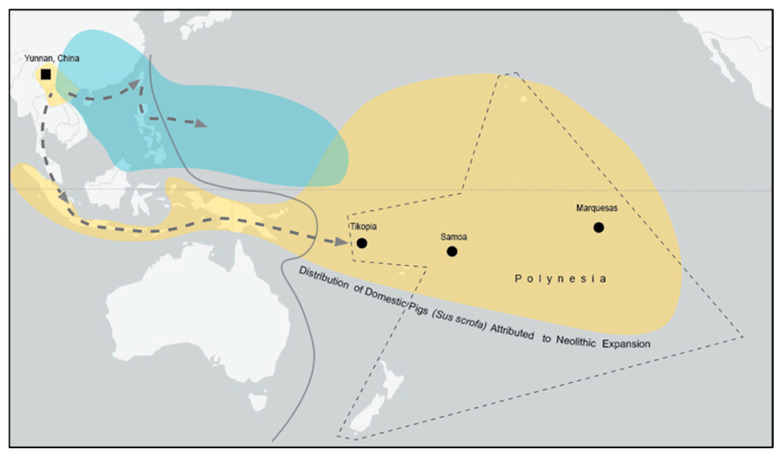
The origins of domesticated pigs in Polynesia. The “Pacific Clade” (yellow) of pig mtDNA is found in northern peninsular Southeast Asia, island Southeast Asia, Near Oceania (west of solid line) and Remote Oceania (east of solid line). Globally, only 43 modern and 9 ancient pigs have been definitively classified as Pacific Clade. This includes examples of domestic and wild/feral pigs. All other Oceanic pigs belong to a separate “East Asian Clade” (blue).

Domestic pigs (*Sus scrofa*), which are found on most islands in Polynesia, belong to a rare mtDNA group referred to as the “Pacific Clade” [3,4,5] (“Mixed Clade 2” in [6]). There is controversy over where the Pacific Clade originated. It was first thought to have come from island Southeast Asia (ISEA) [4], but has more recently been placed in northern peninsular Southeast Asia, based on its discovery in modern pigs in northern Vietnam, northern Laos, and southern China (Yunnan Province) [5]. Some have disputed this claim [7] and argue that the evidence continues to support an ISEA origin for the Pacific Clade (see Figure 1).

We report the first complete mitochondrial genome data for pigs from the islands of the Pacific. Our findings show mtDNA lineages in Polynesia are most closely related to modern wild and domestic pigs in the Yunnan Province of southern China, supporting a northern peninsular Southeast Asia, rather than ISEA, origin for the Pacific Clade. Our results demonstrate the necessity for more complete mtDNA coverage in commensal animals to improve our picture of the settlement of Polynesia (e.g., [8]).

## 2. Materials and Methods

Faunal remains of pigs came from archaeological sites in the Marquesas (Nuku Hiva), Tikopia, and Samoa (‘Upolu) (see Datasets S1 and S2). Twelve ancient pig mtDNA sequences were compared against the pig phylogeny generated by [9] to determine putative haplogroups (see Datasets S3–S5). All ancient DNA lab work was undertaken at a dedicated facility (see Dataset S1 for GenBank accession numbers).

### 2.1. Archaeological Specimens

Faunal remains of pigs came from archaeological sites in the Marquesas (Nuku Hiva) [10], Tikopia [11,12,13], and Samoa (‘Upolu) [14] (Dataset S1). Specimens were clearly identifiable to species based on their form. Samples belong to what is commonly referred to as the Protohistoric period in Polynesia, which includes the last centuries prior to European contact as well as the first centuries after contact. The date of samples was established by radiocarbon dating of deposits, or in the case of the Marquesas specimens, direct AMS dating on bone/tooth. We report these dates in Dataset S2.

### 2.2. Phylogenetic Tree Building

Twelve ancient pig mtDNA sequences were compared against the pig phylogeny generated by [9] to determine putative haplogroups.

The tool AdapterRemoval2 (V.2.3.2) [15] was used to pre-process the raw FASTQ files. This pre-processing step merged the paired-end sequence reads (at least 11 nucleotides overlap). It works by removing short reads (<25 bp), then taking out stretches of Ns and bases with low-quality scores (<30). After pre-processing, collapsed reads were aligned to the complete *Sus scrofa* mitogenome (GenBank accession: EF545567.1) using the BWA aln command [16]. In this step, we employed parameters for ancient DNA. Seeding was disabled (-l 1014) and the frequency of gap opens was set to 2 (-o 2). The maximum edit distance, in this step, was set to 0.03 (-n 0.03). Next, BAM files using DeDup, a tool which has been specifically designed for ancient DNA reads [17], was used to remove PCR duplicates. Damage signatures were assessed using MapDamage2 [18]. Picard’s AddOrReplaceReadGroups was used to add read groups. Excluding reads not mapping to the *Sus scrofa* reference genome was carried out with Samtools. The GATK (V.4.2.3) HaplotypeCaller (Broad Institute, Cambridge, MA, USA) was used for variant calling. To mask regions of coverage regions (minimum coverage 3×), an in-house code was used before the GATK FastaAlternateReferenceMaker. The purpose of this was to generate FASTA sequences for downstream analyses (see Supplementary Materials for GenBank accession numbers).

The variant calls for the ancient Pacific pigs were compared against the pig phylogeny generated by [9] to determine putative haplogroups for the Pacific pigs. It was observed that among the sequences generated from the Pacific pigs, not only was the Pacific Clade present, but so was a few additional but distinct lineages (Dataset S4). Because of this observation of multiple lineages, and the poor coverage of some of the samples (Dataset S3), multiple phylogenetic trees were generated.

Additional *Sus scrofa* complete mitogenomes were downloaded from GenBank (see Dataset S5). These sequences were then compared with samples that had 100% DNA coverage (n = 4) using Mafft (V7). Sequence alignments were visually inspected in Geneious. We made PHYLIP format files for uploading to PhyML 3.0 using SeqMagick [19] (http://www.atgc-montpellier.fr/phyml/, accessed on 14 July 2022) to generate phylogenetic trees, using the *Sus barbatus* mitogenome (Bornean bearded pig; GenBank accession NC_026992.1) as an outgroup. The smart model selection using the Akaike information criterion (AIC) was used [20]. FigTree was used to visualize the output of PhyML.

**Figure 2:**
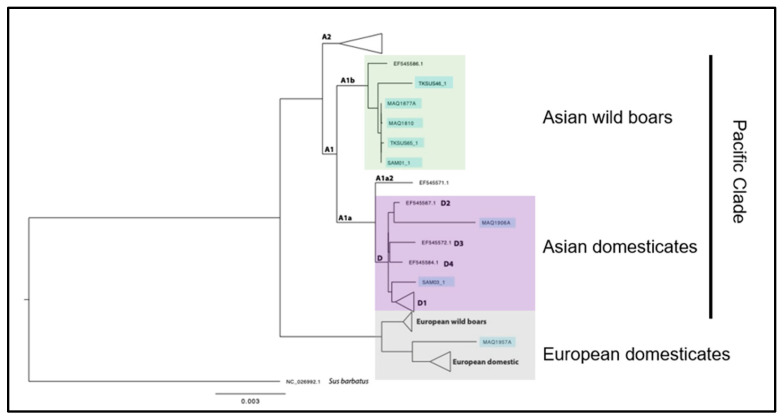
Ancient mtDNA from domestic pigs in Polynesia. We report the first whole genome mtDNA from pigs on Pacific Islands. Our results suggest all founding populations of pigs in Polynesia, first settled by people ca. 2800–700 BP, can be traced back to northern peninsular Southeast Asia. For simplicity, representative samples from GenBank and a sub-set of our results (8 out of 12 specimens) are shown on this phylogenetic tree.

A second tree was constructed using pigs that had at least 90% coverage, using consensus sequences filtered for at least a 3× read depth (see Figure 2). These were aligned using Mafft, and stringent trimming was used to exclude regions where there was missing coverage. This resulted in a concatenated stretch of only 4400 bp shared by 8 of the ancient Pacific pigs. Together with representative sequences for each of the main Asian lineages defined by [9], and all of the mitogenomes from European-sourced pigs in GenBank, a second phylogenetic tree was constructed using the same procedure outlined above. Despite the less than complete mitochondrial genome coverage, this allowed us to explore the genetic relationship of some of the samples that had much worse DNA recovery, but who did have mitochondrial genomes that looked convincingly different than those with 100% coverage (Dataset S4).

### 2.3. Laboratory Protocols for Ancient DNA

DNA was extracted from 22 pig specimens from the Marquesas, Tikopia, and Samoa (see Dataset S1 for details). DNA extraction and the construction of sequencing libraries (Illumina) were undertaken in the ancient DNA laboratory of the Southern Methodist University Molecular Anthropology Laboratories. We extracted DNA following Allentoft et al. [21], with only a minor modification. We substituted a 3M solution for the 5M solution of sodium acetate. Illumina sequencing libraries were prepared using the SRSLY PicoPlus Kit (Claret Bioscience, Santa Cruz, CA, USA) following the manufacturer’s protocol, and quantified on the Bio-Rad CFX96 Touch Real-Time PCR Detection System using the NEBNext Library Quant Kit for Illumina (New England Biolabs, Ipswich, MA, USA). Amplified libraries were visualized under UV, specifically with ethidium bromide on 2% agarose gels. Libraries with only primer and adapter dimers were not further processed. Libraries were enriched for the mitochondrial genome using pig-specific MyBaits probes (MYcroarray, now Arbor Biosciences, Ann Arbor, MI, USA) following the manufacturer’s protocol for low-quantity and low-quality targets, including undertaking two rounds of in-solution capture by hybridization. Libraries were quantified as above, then pooled in equimolar ratios. Finally, they were sequenced on a PE150 S2 NovaSeq6000 flow cell (Maryland Genomics, Baltimore, MD, USA).

## 3. Results

Whole mitochondrial genomes recovered from archaeological samples of domestic pigs from Western Polynesia (Samoa), Eastern Polynesia (the Marquesas), and a Polynesian Outlier (Tikopia) are most closely related to mtDNA lineage A1b, common in Asian wild boars and some domesticates, or D, common to Asian domesticated pigs [9]. One sample clusters with European domestic pigs, and given the radiocarbon date on the sample (1700–1946 cal AD, 2σ, Beta-508540), we believe it represents a 19th C introduction to the Marquesas, rather than the founding population of pigs.

We compared our results with 324 previously sequenced specimens of Sus scrofa. The modern pig lineages most closely related to the newly sequenced ancient Polynesia pigs were both found in southern China’s Yunnan Province (A1b, EF545567.1, and D2, EF545586.1). This is a strong support for a northern peninsular Southeast Asia origin of Pacific Clade pigs.

## 4. Discussion

Geographically widespread genetic studies of the animals that people carried with them on long-distance voyages to Polynesia have become increasingly important in reconstructing Neolithic expansion in the Pacific. Studies of commensal animals hold significant analytical advantages in that these animals have much shorter generation times than people, and unlike plants, they rely heavily on people for long-distance Oceanic dispersal. Animal genetics is also ethically preferable given concerns about individual and group consent raised by studies that reuse blood samples taken for medical trials, or destroy human remains to extract ancient DNA.

Our results suggest that the domestic pigs that were carried out to the islands of Polynesia originated in northern peninsular Southeast Asia. However, much about the routes these pigs took from their putative home remains unclear. Today, Pacific Clade pigs are exceedingly rare in northern peninsular Southeast Asia, and conspicuously absent in southern peninsula Southeast Asia. This may be explained as “a consequence of a replacement of native domestics by pigs later introduced from Central China during several possible demographic expansions of agricultural populations” [5].

The details of pig domestication in China, their global dispersal, and the subsequent interbreeding between pigs from different regions around the world continue to be illuminated through both genetics (e.g., [22]) and archaeology (e.g., [23]). However, our results are inherently limited in scope. Specifically, our whole mtDNA genome phylogenetic trees are not directly comparable to previous trees used to classify mtDNA haplogroups based on partial sequences. We also note that our results are not evidence for the domestication of pigs in Southeast Asia independent of the well-documented domestication of pigs in China, nor are we able to detect introgression of multiple waves of pigs into Polynesia, except for the already documented introduction of European breeds.

The origins and dispersal of the pigs across Polynesia is an excellent example of the advantages, and inherent limitations, of using commensal animals to reconstruct the human past, and the need for better geographic and temporal coverage. Pacific Clade pigs are notably absent from Taiwan—the likely homeland of a portion of the founding human population in Polynesia—and the Philippines. However, new research reports a rare mtDNA lineage closely related to the Pacific Clade in the Philippines [24]. This finding increases the possibility that future studies may uncover multiple dispersal pathways from northern peninsular Southeast Asia to the remote islands of the Pacific.

Looking at commensal animals more broadly, the continued unevenness in geographic coverage for all four animals that were translocated by people around the islands of Polynesia—rat (*Rattus exulans*), pig (*Sus scrofa*), chicken (*Gallus gallus*), and dog (*Canis lupus fimiliaris*)—remains a major roadblock. A recent summary of all reported specimens, a total of 596 individuals (408 modern and 188 ancient animals) [1], noted not only gaps but serious problems of oversampling, with more than 70% of ancient rats (*Rattus exulans*) and 98% of modern chickens (*Gallus gallus*) from a single location.

## 5. Conclusions

Domestic pigs (*Sus scrofa*) were first transported to Polynesia through a series of long-distance voyages ultimately linked to the Neolithic expansion of Austronesian-speaking people out of Asia. The descendants of the founding pigs belong to a rare mtDNA group referred to as the “Pacific Clade” that may have originated in peninsular or island Southeast Asia. We report the first whole genome mtDNA from domestic pigs from any of the remote islands of the Pacific. In this brief report, we describe the close link we discovered between ancient mtDNA from archaeological specimens from across Polynesia and that of modern pigs in northern peninsular Southeast Asia, specifically southern China’s Yunnan Province. More complete mtDNA coverage in commensal animals is necessary to improve our picture of the settlement of Polynesia (ca. 2800–700 years before the present) and specify the route, or routes, that pigs took from northern peninsular Southeast Asia.

## Data Availability

Genetic data available at GenBank. See Database S1 for accession numbers.

## Supplementary Materials

The following supporting information can be downloaded at: https://www.mdpi.com/article/10.3390/ani12182469/s1. The following supporting information: Dataset S1. Archaeological specimens of pig assayed for ancient DNA. Dataset S2. Radiocarbon dates from Marquesas sites. Calibration: BetaCal3.21, HPD method: SHCAL13. Dataset S3. Mapping statistics for ancient Pacific pigs. Dataset S4. Phylogenetic lineage defining SNPs described by [9]. Dataset S5. Complete mitochondrial genomes pulled from GenBank for use in phylogenetic tree. See GenBank for full references.
